# Remarks on Aloes

**Published:** 1885-10

**Authors:** Carroll Dunham


### REMARKS ON ALOES.

Among the remedies of which provings have been published within the last few years, none has seemed to me more deserving of attention than Aloes.

The symptoms which have seemed to me the most characteristic are those of the head and of the abdomen, stool, and urine. They are those on which my use of Aloes in practice has been based. Chief among these are those of the stool.

From these symptoms we gather that Aloes produces a diarrhœa consisting of light-colored simi-liquid fæces, preceded and accompanied by much gurgling and flatus in the abdomen; that the diarrhœa occurs especially in the morning, say from two A. M. to ten A. M.; that the desire for stool is sudden and extremely urgent, being felt in the hypogastrium and in the rectum, and being so urgent that the patient can scarcely retain the fæces long enough to effect the necessary strategic "change of base;" that, during this brief interval, he fears to evacuate wind by the anus or to make any physical exertion, or even to strain to pass water, lest he should have an involuntary evacuation of the bowels. This sensation of the uncertain tenure by which the fæces are held in the rectum is a very well marked characteristic of Aloes, as shown by the following symptoms:

"The evacuation takes place without any exertion on the part of the patient; it seems, as it were, to fall out of the rectum (765). At stool a constant feeling as if there were more fæces to be passed (769). Involuntary passage of fæces when emitting flatus (824). Disposition to stool when passing water (826). Fæces and urine seemed incline to pass and do pass simultaneously (827). When passing water feeling as if a thin stool were about to pass (828). When standing, sensation as if fæces would pass (833)."

There is also a similar frequency or urgency of the desire to pass urine, with a similar uncertainty in the tenure of that excretion, as we perceive from the following symptoms:

"Frequent desire to urinate (990). Increased desire—quantity not increased (992). So urgent a desire he can hardly retain the urine (993). On rising he was obliged to *run quickly* to urinate (996)."

And the similarity of the affection of the urinary organs and the intestines is shown in symptom 1001:

"At stool urination; when urinating desire for stool."

In connection with these two series of symptoms, those of the pelvis deserve notice. Among them we find "heaviness, pressure downward (865, 861). Feeling as if a plug were wedged in between the symphysis pubis and the os coccygis (860)." This is equivalent to a weight upon the perinæum. Viewing it in combination with the symptoms of stool and urine above referred to, we are justified in saying of Aloes, in regard to this portion of its sphere of action, that it strikes the patient equally "between *wind* and *water*."

It is understood, of course, that this is not the only action of Aloes upon the abdominal organs. It is believed, however, to be that variety of action which is most characteristic of the remedy, and the least likely to be confounded with the effect of any other drug. In the frequent desire for stool; in the frequent, pappy, not very abundant stool; in the pressure downward in the back and pelvis; in the abundant formation of flatus in the abdomen which rumbles and gurgles, producing pinching pain in the lower part of the abdomen just before the stool, the action of Aloes very closely resembles that of Nux vom., a remedy so useful in diarrhœa and dysentery. It is distinguished, however, by the peculiarities of the evacuation of stool. Nux vom. produces very frequent desire for stool, with inability to evacuate the fæces. Under Aloes, on the contrary, the difficulty is to retain the fæces as long as the patient desires to do so. Aloes seems to paralyze the sphinctor ani to a certain extent; Nux vom. to excite it in a spasmodic action of exalted power. In this action on the sphincter, Aloes resembles Hyoscyamus.

Among the symptoms of the head I am inclined to regard as characteristic of Aloes those which describe a heavy, confused dullness in the front part of the head extending to the root of the nose, with inability to think; a pain in the forehead which compels the patient to close the eyes, or, if he wishes to look at anything, to constringe the eyes, making the aperture of the lids very small. It must be admitted, however, that symptoms so similar to these are found under other remedies, that these symptoms *alone* could not be regarded as a sure indication for Aloes.

The following cases will show how I have prescribed Aloes, and will suggest some reflections upon the mode of selecting remedies in practice.

Within the last three years I have treated about thirty-five cases which so closely resemble each other in their characteristic elements that the description of all may be given in that of the last of the series, which came under my care a month ago.

A young man applied for relief from a diarrhœa which had persisted about two weeks in spite of various remedies which had been prescribed for it, and among which were Calcarea, Nux vom., Bryonia, and the inevitable Arsenicum. He described his stools as being light yellow, pappy, somewhat frothy, and tolerably abundant. They were preceded by flatulent rumbling in the abdomen and by pinching pain in the hypogastrium. The necessity for a stool awakened him from a sound sleep about three A. M. From this hour to nine A. M. he had from four to six stools of the character above described. None at any other period of day or night. When the desire for stool was felt, the urgency became instantly so great that he was compelled to spring from the bed and hasten to the water-closet. Yet this urgency *was not of the nature of tenesmus*, but *rather a sensation* of weakness in the sphincter, as though he could not prevent the fæces from falling out. During stool, which passed freely, in a mass, the instant the restraint of the patient's volition was withdrawn from the sphincter ani there was a slight burning in the rectum. After stool, cessation of pain, but a very slight general sensation of weakness and lassitude.

During this period, from three to nine A. M., the patient was compelled to avoid all rapid or severe exertion of body, and especially straining to pass water. The penalty of such exertion or straining was sure to be an involuntary evacuation of fæces.

I prescribed one powder of Sac. lactis containing two globules of Aloes$^{200}$, to be taken dry on the tongue at ten A. M. (the hour at which he called on me). From this time he had no diarrhœa. The next morning he slept until seven A. M., and at nine had a natural stool, as was his habit in health.

### Case II.

During the winter season a gentleman, about seventy years of age, applied for relief from a dull, heavy frontal headache, which incapacitated him from mental labor. He could give me no more definite nor characteristic description of his ailment. It was felt as soon as he waked, and lasted all day. From such a description as the above, it would be impossible to prescribe with any certainty of selecting the right remedy. I set myself therefore to investigate the patient's previous history, in the hope of getting some help from the Anamnesis, to which Hahnemann and Bœnninghausen attach so much importance. I learned that this headache was no new affliction. It had for years annoyed this gentleman, rather more during the winter season, whereas during the summer he was comparatively free from it. No peculiarity of diet or regimen could explain this fact.

On the other hand, I learned that during the summer season my patient was very frequently attacked with diarrhœa, the disease coming on suddenly, waking him at two A. M., with a pinching flatulent colic, and so urgent a call to evacuate the bowels that he would be compelled to seek the water-closet instantly, experiencing meanwhile the greatest difficulty in retaining the fæces. From this time till ten A. M. he would have four or five stools, pappy, copious, light yellow, great difficulty in retaining the fæces for even a moment after the desire for stool was first experienced. Desire for stool provoked by eating, so that he was compelled to leave the breakfast table. Involuntary stool when straining to pass water. When comparatively free from headache, he was inclined to diarrhœa, and *vice versa.*

I have long been persuaded that a most important condition of success in the treatment of chronic diseases consists in the practitioner taking such a view of the case as shall combine the various ailments of which a chronic patient may complain at different periods of time and in different organs, even though these periods and organs be remote from each other and apparently disconnected. In no other way, it has sometimes seemed to me, could the characteristic indications of the remedy for such a case be found.

Acting upon this persuasion in the case in question, I regarded the headaches which predominated in winter and the diarrhœas which predominated in summer as, in some sort, complementary series of symptoms, and as making up, both together, the "totality of symptoms" for which I was to seek, in the *Materia Medica*, the simillimum.

The symptoms of the headache, indeed of the entire winter affection, presented nothing that was characteristic of any one remedy to the exclusion of all others. Carbo veg., Sabadilla, Sulphur, Aloes, Nux vomica, and several others might be regarded as about equally well indicated.

When, however, to the head symptoms of the winter, I came to add the diarrhœa symptoms of the summer, regarding the *sum total* as *one* disease, it was then impossible to avoid perceiving that the diarrhœa symptoms were strikingly characteristic of Aloes, and could not indicate any other remedy. This furnished the clue to the prescription. On studying the head symptoms of Aloes, it was seen that they corresponded to the head symptoms of my patient quite as well as the symptoms of any other drug. Aloes$^{200}$ was given and it afforded a relief which my patient had sought in vain from other remedies taken on the strength of the head symptoms alone. The headache returned a few times afterward with very much diminished severity, but yielded at once to Aloes. Latterly my patient has been entirely free from it, nor did the diarrhœa return as it used formerly to do whenever the headache ceased to prevail.

In a third case I have given Aloes for incontinence of urine in an old gentleman who has enlarged prostate. The prescription was based on the fact that he is very subject to a diarrhœa, presenting all the characteristics of the Aloes diarrhœa. The peculiarities of the incontinence, moreover, correspond to those of the Aloes urine symptoms. Thus far the success of the treatment leaves nothing to desire. But as the patient has been but a few weeks under the treatment, it is too soon to express a decided judgment or to entertain sanguine expectations of a cure.

CARROLL DUNHAM.

